# All Akt Isoforms (Akt1, Akt2, Akt3) Are Involved in Normal Hearing, but Only Akt2 and Akt3 Are Involved in Auditory Hair Cell Survival in the Mammalian Inner Ear

**DOI:** 10.1371/journal.pone.0121599

**Published:** 2015-03-26

**Authors:** Yves Brand, Soledad Levano, Vesna Radojevic, Arianne Monge Naldi, Cristian Setz, Allen F. Ryan, Kwang Pak, Brian A. Hemmings, Daniel Bodmer

**Affiliations:** 1 Department of Biomedicine, University Hospital Basel, Hebelstrasse 20, 4031 Basel, Switzerland; 2 Clinic for Otolaryngology, Head and Neck Surgery, University Hospital Basel, Petersgraben 4, 4031 Basel, Switzerland; 3 Inner Ear Research, Clinic for Otolaryngology, Head and Neck Surgery, University Hospital Zürich, Frauenklinikstrasse 24, NORD 2, 8091 Zürich, Switzerland; 4 Departments of Surgery/Otolaryngology, UCSD School of Medicine, 9500 Gilman Drive MC0666, La Jolla, CA 92093, United States of America; 5 Department of Neurosciences, UCSD School of Medicine, 9500 Gilman Drive MC0666, La Jolla, CA 92093, United States of America; 6 San Diego VA Medical Center, 3350 La Jolla Village Drive, San Diego, CA 92161, United States of America; 7 Mechanisms of Cancer Program, Friedrich Miescher Institute for Biomedical Research, Basel, Switzerland; Universitat Pompeu Fabra, SPAIN

## Abstract

The kinase Akt is a key downstream mediator of the phosphoinositide-3-kinase signaling pathway and participates in a variety of cellular processes. Akt comprises three isoforms each encoded by a separate gene. There is evidence to indicate that Akt is involved in the survival and protection of auditory hair cells *in vitro*. However, little is known about the physiological role of Akt in the inner ear—especially in the intact animal. To elucidate this issue, we first analyzed the mRNA expression of the three Akt isoforms in the inner ear of C57/BL6 mice by real-time PCR. Next, we tested the susceptibility to gentamicin-induced auditory hair cell loss in isoform-specific Akt knockout mice compared to wild-types (C57/BL6) *in vitro*. To analyze the effect of gene deletion *in vivo*, hearing and cochlear microanatomy were evaluated in Akt isoform knockout animals. In this study, we found that all three Akt isoforms are expressed in the cochlea. Our results further indicate that Akt2 and Akt3 enhance hair cell resistance to ototoxicity, while Akt1 does not. Finally, we determined that untreated Akt1 and Akt2/Akt3 double knockout mice display significant hearing loss, indicating a role for these isoforms in normal hearing. Taken together, our results indicate that each of the Akt isoforms plays a distinct role in the mammalian inner ear.

## Introduction

Akt is a serine/threonine kinase that serves as an important mediator of various cellular processes [1]. It is one of the key downstream mediators of the phosphoinositide-3-kinase (PI3K) signaling pathway, which can be activated through cell-surface receptors by a number of signaling molecules and leads to activation of Akt by phosphorylation [2,3,4,5,6,7]. A major downstream effector of Akt is nuclear factor-kappaB (NfκB), which can link Akt signaling to the cell nucleus. NfκB also appears to feed back on Akt activation, since its inhibition leads to a reduced pAkt/Akt ratio [8].

There are three isoforms of Akt: Akt1, Akt2 and Akt3 [9,10,11,12,13,14]. These isoforms are encoded by different genes, but share a conserved domain structure consisting of an N-terminal pleckstrin homology domain, a kinase domain and a C-terminal regulatory domain containing a hydrophobic motif [13]. Akt1 is ubiquitously expressed, while Akt2 is primarily expressed in insulin-responsive tissues and Akt3 is highly expressed in brain and testes [15]. Better understanding of Akt signaling has been obtained through *in vivo* studies using transgenic and knockout models [16,17]. As a result, each isoform has been linked to specific physiological functions [18].

Sensorineural hearing loss is linked to degeneration and death of the auditory hair cells (HCs) and their associated spiral ganglion neurons. In mammals, loss of HCs is irreversible. A better understanding of the survival pathways and molecular events involved in protection of the auditory epithelium is therefore essential for developing therapeutic strategies to prevent hearing loss. Activated Akt has been detected in several inner ear structures indicating that Akt plays a role in inner ear physiology [19]. While the role of Akt itself has not been well studied in the inner ear, it was found that PI3K signaling mediates HC survival and opposes gentamicin toxicity *in vitro* [20]. Moreover, studies of NfκB have demonstrated that NfκB inhibition leads to HC loss *in vitro*, and this process involves changes in PI3K signaling [21,22]. Furthermore, NfκB activation has been shown to protect HCs from aminoglycoside-induced damage [23].

While the above evidence implicates Akt in the inner ear, its physiological role remains unknown. Moreover, the participation of Akt isoforms in the inner ear has yet to be explored. To address these issues, we analyzed mRNA expression of all Akt isoforms in the cochlea of C57/BL6 mice by real-time PCR. Next, we tested the susceptibility on gentamicin-induced auditory HC loss in isoform specific Akt knockout mice *in vitro*. Finally, to analyze the effect of gene deletion in the intact animal, hearing and cochlear microanatomy were evaluated in isoform-specific Akt knockout mice.

## Materials and Methods

### Animal procedures

All animal procedures were carried out according to an approved animal research protocol (Kantonales Veterinäramt, Basel, Switzerland). For tissue extraction and culture, 5-day-old C57/BL6 pups (Harlan, Netherlands) and 5-day-old Akt1^-/-^, Akt1^+/-^, Akt2^-/-^, Akt2^+/-^, Akt3^-/-^, Akt3^+/-^ and Akt2^-/-^ Akt3^-/-^ mice were used. For the auditory brainstem response (ABR) audiometry, 3–4 month-old wild-type and knockout (Akt1^-/-^, Akt2^-/-^, Akt3^-/-^, Akt2^-/-^ Akt3^-/-^) mice were available.

#### Akt knockout model

Generation of transgenic animals was carried out in the Friedrich Miescher Institute for Biomedical Research, Basel, Switzerland. Akt1, Akt2 and Akt3 knockout mice were generated as described elsewhere and backcrossed to a C57/BL6 background [17,24,25]. Genotyping was carried out to verify the genetic identity of the pups using multiplex PCR.

### Tissue extraction and culture

For tissue extraction and culture, 5-day-old C57/BL6 wild-type and 5-day-old Akt1^-/-^, Akt1^+/-^, Akt2^-/-^, Akt2^+/-^, Akt3^-/-^, Akt3^+/-^, Akt2^-/-^ Akt3^-/-^ pups were decapitated, and cochlear microdissections were performed under a light microscope to isolate the organ of Corti (OC), the spiral ganglion (SG), and the stria vascularis (SV). Brain tissue was removed from the same animals. During the microdissection, the different tissues were maintained in ice-cold PBS.

For experiments in which organ cultures were needed, OCs were incubated in culture medium consisting of Dulbecco’s Modified Eagle Medium, 10% fetal calf serum, 25 mm HEPES and 30 U/ml penicillin (Invitrogen, Carlsbad, USA) for 24 hours at 37° C, 5% CO_2_. To analyze HC damage OCs were incubated with or without 0.5 mm gentamicin (Sigma-Aldrich, St. Louis, USA) for 24 hours.

#### RNA extraction

Total RNA was isolated from a pool of 20 OCs, SGs, SVs and from 20–40 mg brain of 5-day-old C57/BL6 mice pups using the RNAeasy Minikit (Qiagen, Hombrechtikon, Switzerland) including DNase treatment according to the supplier’s instructions. To homogenize the tissues, we used the Ultra-Turrax T8 homogenizer (IKA-Werke, Staufen, Germany). The quantity and quality of the isolated RNA was determined with a NanoDrop ND 1000 (NanoDrop Technologies, Delaware, USA). The 260/280 nm ratio of all our samples was between 1.8 and 2.1.

#### Real-time PCR

Gene sequences from Akt1 (NM_001165894.1), Akt2 (NM_007434.3), and Akt3 (NM_011785.3) were accessed from GenBank. Primers were designed using the Universal ProbeLibrary Assay Design Center (Roche Applied Biosciences, Rotkreuz, Switzerland).

Total RNA (500 ng) was reverse transcribed into cDNA with a first strand cDNA synthesis kit (Roche Applied Biosciences) according to supplier’s instructions. The amplification reaction took place in an ABI Prism 7900HT Sequence Detection System (Applied Biosystems) using a Fast Start Universal SYBR Green Master (Rox) (Roche Applied Biosciences Foster City, USA). The primer sequences were: Akt1 forward, 5′-tcgtgtggcaggatgtgtat-3′, Akt1 reverse 5′-acctggtgtcagtctcagagg-3′, Akt2 forward 5′-cagctgggagacccaaga-3′, Akt2 reverse 5′-cacacgctgtcacctagctt-3′, Akt3 forward, 5′-tggaccactgttatagagagaacattt-3′, Akt3 reverse 5′-tggatagcttccgtccactc-3′, (Microsynth, St. Gallen, Switzerland). Each reaction contained 300 nm of primer. The cycling parameters were 10 minutes at 95°C, then 40 cycles of 15 seconds at 95° C and 60 seconds at 60° C. We calculated relative quantities of specifically amplified cDNA with the comparative threshold cycle method. GAPDH acted as an endogenous reference (Microsynth). The central nervous system has been reported to express all Akt isoforms [26]. Therefore, brain tissue was used as positive control in real-time PCR. No-template and no-reverse-transcription controls ensured that nonspecific amplification and DNA contamination could be excluded.

#### Hair cell counts

OCs from 5-day-old wild type-mice (n = 17) and OCs from 5-day-old knockout mice were used, Akt1^-/-^ (n = 1), Akt1^+/-^ (n = 9), Akt2^-/-^ (n = 6), Akt2^+/-^ (n = 9), Akt3^-/-^ (n = 5), Akt3^+/-^ (n = 18), Akt2^-/-^ Akt3^-/-^ (n = 12). The OCs were fixed in 4% paraformaldehyde containing PBS and permeabilized with 5% Triton X-100 in PBS containing 10% fetal calf serum. OCs were incubated with a 1:100 dilution of Texas Red X-phalloidin (Molecular Probes, Eugene, USA) for 45 minutes at room temperature. We visualized the OCs using a fluorescence microscope (Olympus IX71) and photographed with an AxioCam (Zeiss, San Diego, USA). Surviving HCs were counted in a section corresponding to 20 inner HCs at three different sites located on the basal and middle turns of each OC. Surving of HCs was quantified including surviving inner and outer HCs. The rational to inclued inner HC survival to our quantification is the fact that significant inner HC death was observed in some of the gentamicin-treated samples. HC counts were analyzed by analysis of variance (ANOVA) followed by the least significant difference (LSD) post-hoc test with Bonferroni correction for multiple tests (Stat View 5.0). Differences associated with corrected p-values of less than 0.05 were considered to be statistically significant. All data are presented as mean ± standard deviation.

#### ABR audiometry

Wild-type and Akt knockout mice used for ABR measurements were three to four months of age. Mice had the following genotypes, wild-type (n = 11), Akt1^-/-^ (n = 5), Akt2^-/-^ (n = 8), Akt3^-/-^ (n = 10) and Akt2^-/-^/Akt3^-/-^ (n = 8). Auditory sensitivity was assessed with ABR thresholds obtained with a TDT System 3 auditory evoked potential workstation running BioSigRP software (Tucker-Davis Technologies, Alachua, FL, USA). Mice were anesthetized with an intraperitoneal injection of a mixture of ketamine and xylazine (0.12/0.01 mg/g body weight). Potentials were recorded with subcutaneous stainless-steel electrodes as the potential difference between an electrode on the vertex and an electrode on the mastoid, whereas the lower back served as ground. ABR waveforms were recorded with a TDT RA4LI low-impedance digital headstage and a RA4PA Medusa preamp controlled by an RA16 digital signal processor (Tucker-Davis Technologies), and averaged in response to 500 click stimuli. The stimuli (0.1-ms duration, 10/s rate) were delivered through a closed acoustic system and were calibrated using a sound level meter (precision integrating sound level meter type 2218; Brüel & Kjaer, Naerum, Denmark) and a 2CC coupler. Clicks were reduced in intensity from 80 dB sound pressure level (SPL) in 5-dB steps. The hearing threshold was defined as the lowest intensity that induced the appearance of a visually detectable peak. Results are presented as the mean ± standard deviation, and statistical analysis was performed using two-way ANOVA (Stat View 5.0). Significance was determined as *p* < 0.05.

#### Histology

Mice were sacrificed immediatly after ABR audiometry with an overdose of sodium pentobarbital (100 mg/kg) and transcardially perfused with 50 ml of phosphate-buffered 4% paraformaldehyde (pH 7.4, at 4°C). The inner ear was carefully removed. Decalcification was carried out in a light-protected flask for 10 days in a solution of 120 mM EDTA (Merck, New Jersey, USA) in distilled water (pH 6.8). After decalcification, cochleae were prepared for paraffin embedding. Briefly, cochleae were dehydrated in graded ethanol solutions (at 70%, 80%, 95%, and 3 × 100%, each for 1 h; 3 × xylol for 1 h; 2 × paraplast at ‒60°C for 1 h; and paraplast at −60°C for 10 h), and embedded in paraffin at 65°C.

For histological evaluation, cochlear sections of 8 μm thickness were cut on a Leitz microtome and mounted on Superfrost plus slides (Menzel, Braunschweig, Germany). Sections were deparaffinized, rehydrated, washed in PBS for 5 min. and stained with hematoxylin and eosin.

Sections were visualized on an Olympus AX-70 microscope equipped with a spot digital camera. Recorded images were adjusted for brightness and contrast using Image-Pro Plus and Photoshop image processing software.

## Results

### Akt1 and Akt3 mRNA are homogenously expressed in the cochlea, while Akt2 gene expression is higher in the OC and the SV than in the SG

**Fig 1:**
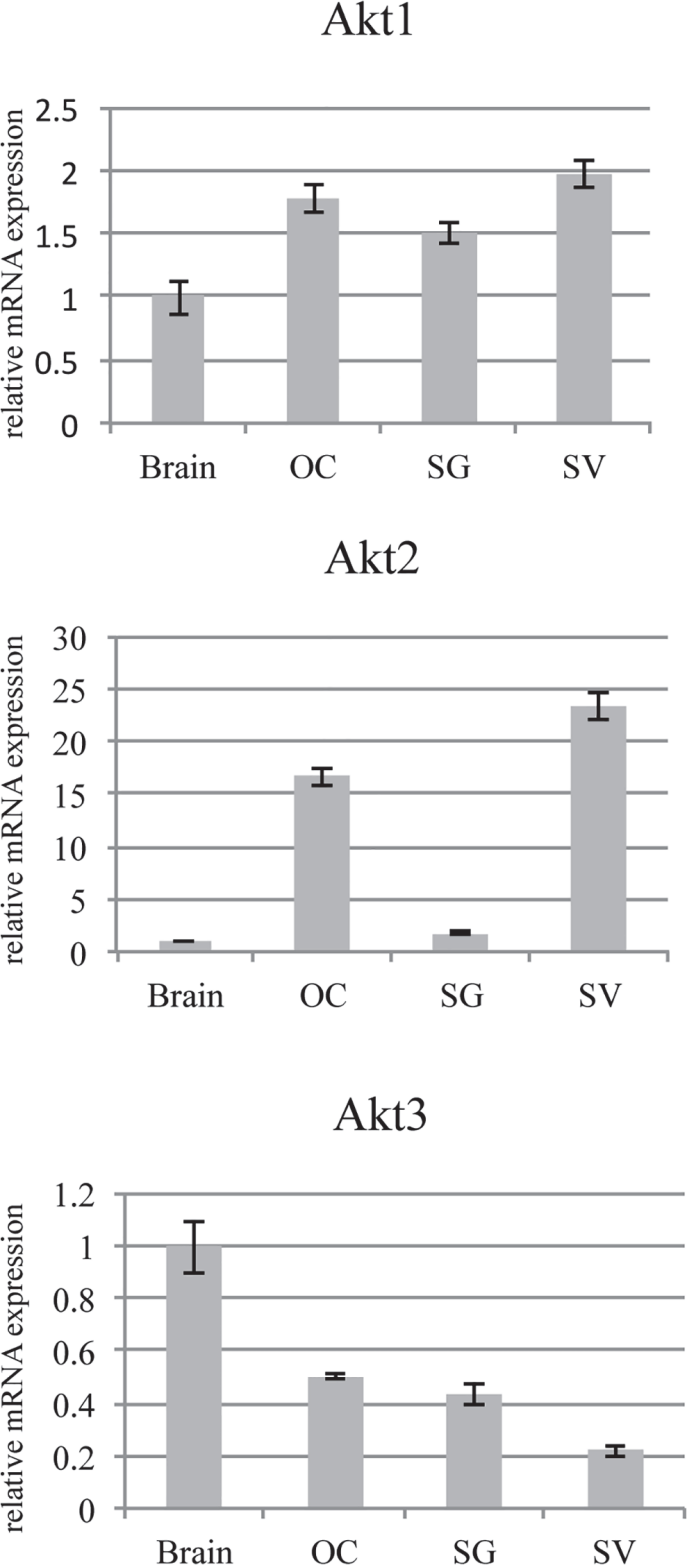
Akt1, Akt2 and Akt3 mRNA relative expression in the organ of Corti (OC), the spiral ganglion (SG) and the stria vascularis (SV) of 5-day-old C57/B6 mice. Expression was measured by quantitative real-time PCR with GAPDH as an endogenous control. Akt1, Akt2 and Akt3 expression levels are presented relative to expression in the brain. Each bar represents mean ± standard deviation. Each experiment was repeated three times, with different biological replicates in triplicate. For each experiment mRNA of 20 ears were pooled.

The gene expression of Akt isoforms were determined in cochlear tissues of 5-day-old C57/BL6 mice pups using real-time PCR. Brain tissue was used as a comparative mRNA standard. The expression levels of Akt1 and Akt3, relative to brain, were nearly equally distributed across the three cochlear tissues. The relative gene expression of Akt1 in the cochlea was 1.5 to 2 times higher than in brain, while the relative gene expression of Akt3 in the cochlea was 0.2 to 0.5 of the level observed in brain tissue. In contrast, Akt2 relative mRNA levels were much higher in the OC and SV than in the SG (p<0.001 and p<0.001 respectively). Akt2 relative gene expression was over 16-fold higher in the OC and over 23-fold higher in the SV than in brain tissue. Akt2 relative gene expression in the SG was only 1.8 times higher than in brain tissue (Fig. 1).

### OC explants from Akt2^+/-^, Akt2^-/-^,Akt3^+/-^,Akt3^-/-^ and Akt2^-/-^ Akt3^-/-^ double knock out mice show increased susceptibility to gentamicin-induced HC loss while OC explants from Akt1^+/-^ and Akt1^-/-^ mice do not show increased susceptibility to gentamicin-induced HC loss compared to their wild-type littermates *in vitro*

**Fig 2:**
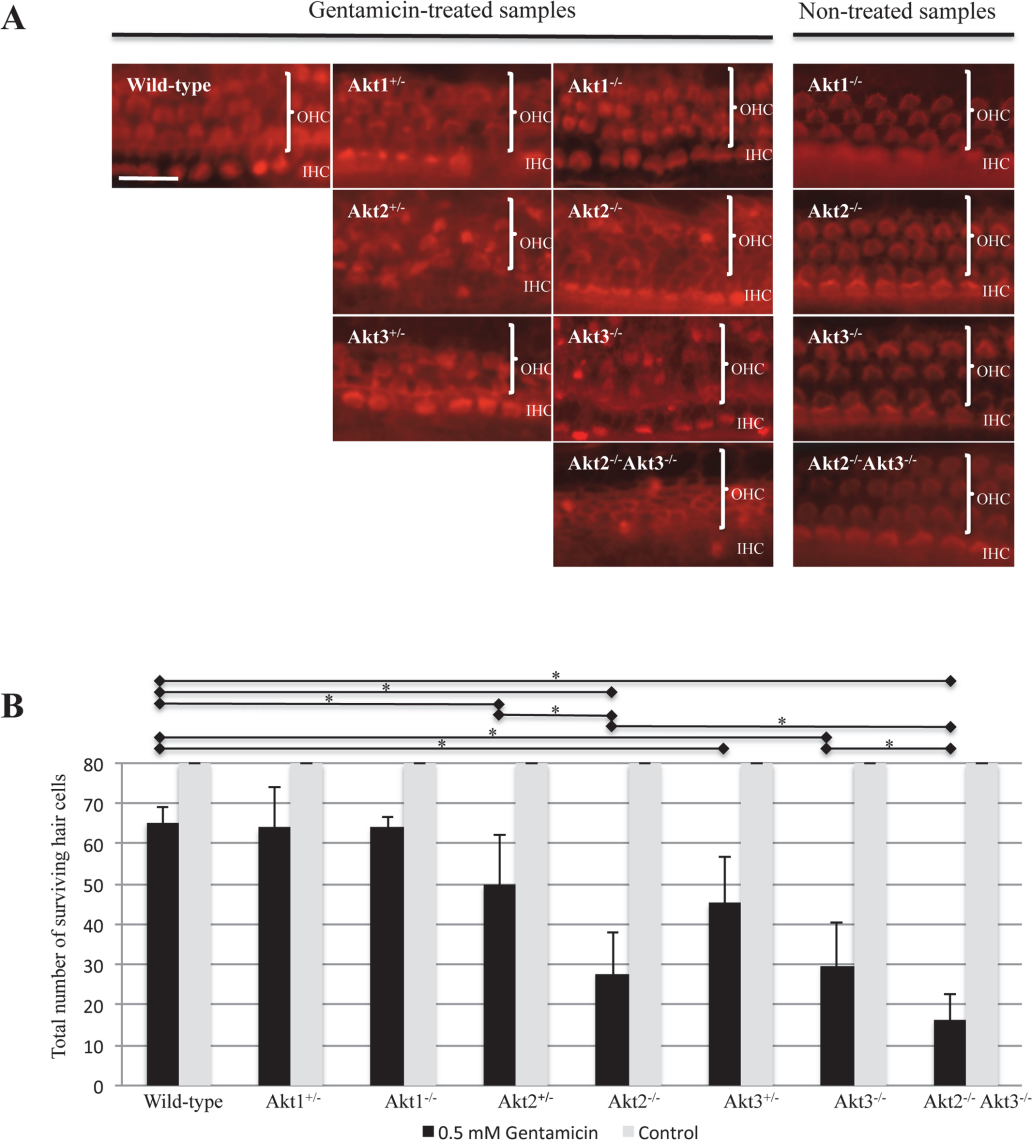
A) Gentamicin-induced hair cell (HC) damage in cultured neonatal OC explants from Akt1, Akt2, Akt3 single and double knockout mice. Micrographs illustrate phalloidin-labeled organs of Corti (OCs). OCs were exposed to 0.5 mM gentamicin for 24h. Akt1^+/-^ and Akt1^-/-^ mice show no additional HC loss compared to wild-type littermates. Akt2^+/-^ and Akt3^+/-^ mice show additional HC loss compared to wild type mice. Akt2^-/-^ and Akt3^-/-^ knockout mice show additional increased HC loss, with only a small number of outer HC (OHC) remaining compared to their heterozygous littermates. The most pronounced HC loss is seen in Akt2^-/-^ Akt3^-/-^ knockout mice, where only a few OHCs survive and a large number of inner HCs (IHCs) are lost. The three OHC rows and the single inner HC row can be well recognized in OCs untreated with gentamicin. Genotypes are described as wild-type, heterozygous (+/-) and homozygous (-/-). Scale bar = 20 μm. **B)** Quantitative analysis of surviving hair cells (HCs) after gentamicin treatment *in vitro*. OCs were exposed to 0.5 mM gentamicin for 24h. Each bar represents mean ± standard deviation. *Asterisks* indicate a significant (p<0.05) difference between the indicated groups. (Numbers of OC explants analyzed: Wild-type n = 17, Akt1^-/-^ n = 1, Akt1^+/-^ n = 9, Akt2^-/-^ n = 6, Akt2^+/-^ n = 9, Akt3^-/-^ n = 5, Akt3^+/-^ n = 18, Akt2^-/-^ Akt3^-/-^n = 12).

We analyzed the susceptibility to gentamicin-induced HC loss of 5-day-old Akt1^+/-^,Akt1^-/-^,Akt2^+/-^, Akt2^-/-^,Akt3^+/-^,Akt3^-/-^ and Akt2^-/-^ Akt3^-/-^ double knock out mice and their wild-type littermates *in vitro* (Fig. 2). OC explants were incubated in culture medium with the presence of 0.5 mm gentamicin for 24 hours. Controls were treated without the presence of 0.5 mm gentamicin in the culture medium. HC loss was quantified in the basal and middle turns of each OC.

Akt2^+/-^ (p = 0.0028), Akt2^-/-^ (p>0.001), Akt3^+/-^ (p = 0.0005), Akt3^-/-^ (p<0.001) and Akt2^-/-^ Akt3^-/-^ double knock out (p<0.001) mice showed increased susceptibility to gentamicin-induced HC loss compared to their wild-type littermates *in vitro*. Moreover, Akt2^-/-^ mice showed significantly increased HC loss compared to Akt2^+/-^ mice treated with gentamicin *in vitro* (p = 0.0042). In contrast, there was no significant difference in gentamicin-induced HC loss between Akt3^-/-^ and Akt3^+/-^ mice (p = 0.0638). Next, we analyzed the susceptibility to gentamicin-induced HC loss of 5-day-old Akt2^-/-^ Akt3^-/-^ double knockout mice compared to Akt2^-/-^ and Akt3^-/-^ single knock out mice. Akt2^-/-^ Akt3^-/-^ double knock out mice showed increased susceptibility to gentamicin-induced HC loss compared Akt2^-/-^ and Akt3^-/-^ single knock out mice to *in vitro* (p = 0.0203 and p<0.0177, respectively).

There is no significant difference in gentamicin-induced HC loss between Akt1^-/-^, Akt1^+/-^ and wild-type mice. No HC loss was observed when OC explants were treated with culture medium without gentamicin.

### Akt1^-/-^ and Akt2^-/-^Akt3^-/-^ mice show elevated ABR thresholds compared to wild-type littermates

**Fig 3:**
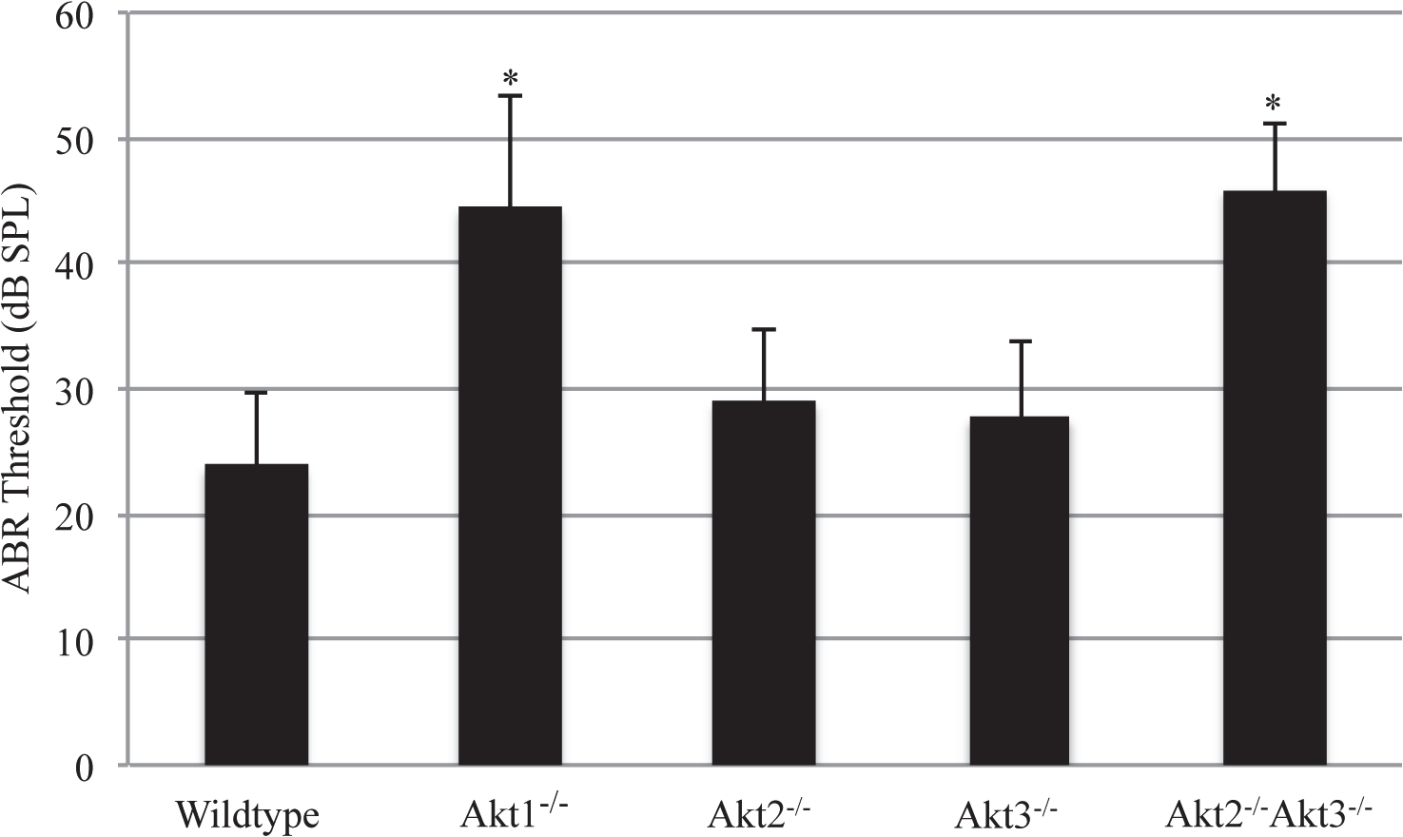
Click-induced ABR thresholds of wild-type versus Akt knockout mice at 3–4 months of age. Normal hearing threshold was about 25 dB SPL. The ABR thresholds of single Akt1^-/-^ knockout and Akt2^-/-^/Akt3^-/-^ double knockout mice were significantly higher than their corresponding wild-types (* p<0.05). Bars represent mean ± standard deviation. (Numbers of mice measured: n = 11 for wild-type, n = 5 for Akt1^-/-^, n = 8 for Akt2^-/-^, n = 10 for Akt3^-/-^, n = 8 for Akt2^-/-^ Akt3^-/-^).

Hearing thresholds were determined by click-induced ABR in Akt1^-/-^, Akt2^-/-^, Akt3^-/-^, Akt2^-/-^ Akt3^-/-^ and wild-type littermates at 3–4 months of age (Fig. 3). Akt1^-/-^ mice (44.5±8.9 dB SPL) and Akt2^-/-^Akt3^-/-^ mice (45.9±5.3 dB SPL) exhibited elevated ABR threshold compared to their wild-type littermates (24.3±5.7 dB SPL) (p = 0.0008 and p<0.0001, respectively). There were no differences in ABR thresholds of Akt2^-/-^ and Akt3^-/-^ mice compared to their wild-type littermates. No differences were observed in ABR waveforms and interpeak latencies between the different genotypes (data not shown).

### Akt1^-/-^, Akt2^-/-^, Akt3^-/-^, Akt2^-/-^Akt3^-/-^ and wild-type mice show similar cochlear microanatomy

**Fig 4:**
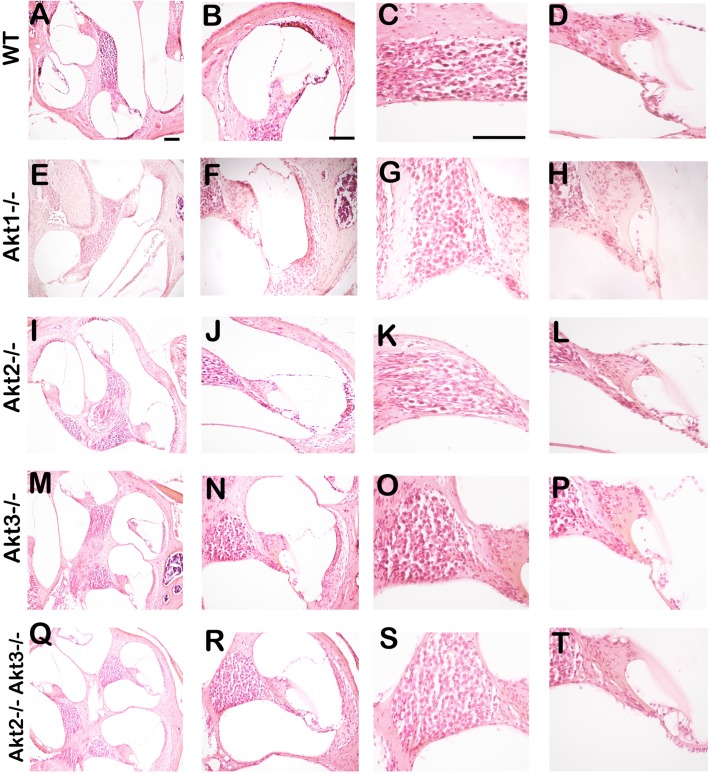
Light microscopy images of Akt single and double knockouts as well as wild-type (WT) mouse cochleas (sagittal section) stained with hematoxylin/eosin. No differences were observed between the morphology of the single and double knockout mice compared with WTs. Arrows and abbreviations indicate the location of the inner HC (IHC), the outer HC (OHC), tectrial membran (TM) SG neuron (SGN) and the stria vascularis (SV). Scale bar = 100 μm (A, E, I, M, Q); Scale bar = 100 μm (B, F, J, N, R); Scale bar = 100 μm (C, D, G, H, K, L, O, P, S, T).

Sections from the temporal bones of Akt1^-/-^, Akt2^-/-^, Akt3^-/-^, Akt2^-/-^Akt3^-/-^ and wild-type mice were stained with HE to study possible differences in cochlear microanatomy. As revealed by microscopy, there were no significant differences in the microanatomy between different Akt knockout and the wild-type mice (Fig. 4). All display an OC with 3 rows of outer HCs and 1 row of inner HCs (Fig. 4D, 4H, 4L, 4P, 4T). The morphology of the tectorial (Fig. 4D, 4H, 4L, 4P, 4T) and basilar membranes (Fig. 4B, 4F, 4J, 4N, 4R), the spiral ganglion neurons (Fig. 4C, 4G. 4K 4O, 4S), and the stria vascularis (Fig. 4B, 4F, 4J, 4N, 4R) were similar.

## Discussion

The purpose of the present study was to investigate the role of Akt isoforms in the inner ear, with a focus on HC survival. Initially, we documented the expression levels of the three Akt isoform mRNAs in the inner ear. Second, we determined the gentamicin susceptibility of OC explants from Akt isoform knockout mice *in vitro*. Finally, we determined the hearing levels and assessed the cochlear microanatomy in these mice to elucidate the physiological role of Akt in the intact animal.

Relative mRNA expression for Akt1 and Akt3 compared to brain tissue was roughly similiar in the OC, SG and SV for Akt1 and Akt3. In contrast, Akt2 is expressed primarily in the OC and SV, at levels 16-fold higher in the OC and 23-fold higher in the SV when compared to brain tissue, respectively.

From previous studies of Akt isoform-specific knockout mice we know that the different Akt isoforms have different functions depending on tissue and extracellular stimulus. Akt1 knockout animals are smaller than their wild-type littermates and Akt1^-/-^ cells display higher rates of apoptosis [18]. Akt2 knockout mice develop a type 2 diabetes-like phenotype and therefore it has been speculated that Akt2 plays a central role in glucose homeostasis [27]. Finally, Akt3 knockout mice display impaired brain development and therefore, it has been proposed that Akt3 plays a role in brain development [25]. However, it should be noted that expression level alone does not dictate the importance of a given isoform. Different mechanisms have been proposed to contribute to Akt isoform distinctions, among them defined tissue distribution, differential activation by extracellular stimuli, distinct intrinsic catalytic activity, and cell context specific factors including subcellular compartmentalization [28].

Several studies in Akt1^-/-^, Akt2^-/-^, Akt3^-/-^ mice show no compensatory upregulation of the remaining isoforms (assessed at the mRNA and protein level) [27,29,30]. Moreover, there was no compensatory upregulation of Akt1 in Akt2^-/-^Akt3^-/-^ mice [17]. In all these mutant mice, there was a significant reduction of total phosphorylated/activated Akt. This indicates mice lacking one Akt isoform are not able to fully compensate for the lack of the other isoforms. Compensatory upregulation was extensivly studied in several systems, including the central nervous system. Given the close homolgy between the central nervous system to the auditory system, we can only speculate that no compensatory upregulation takes place in the inner ear in Akt mutant mice. This is in line with our observation that Akt1 is not able to compensate for the lack of the other isoforms in gentamicin-induced HC loss and no compensatory effects in gentamicin-induced HC loss was observed in mice lacking either the Akt2 or Akt3 isoform. Elevated ABR thresholds in Akt1^-/-^ indicates that Akt2 or Akt3 can not compensate fo the lack of Akt1 while elevated ABR thresholds in Akt2^-/-^Akt3^-/-^ mice shows that Akt1 can not compensate for the lack of Akt2 and Akt3. However, Akt2^-/-^ and Akt3^-/-^ mice show normal ABR thresholds while thresholds in Akt2^-/-^Akt3^-/-^ mice are elevated. Therefore, compensatory mechanisms still have to be considered. However, compenstaory upregulation of the remaining Akt isoforms have not been observed so far and overlapping roles for Akt2 and Akt3 is another explanation for the effects observed.

Cell survival signaling pathways suppress the intrinsic cell death machinery and thereby prevent apoptosis. In recent years, several cell survival pathways have been characterized, among them the PI3K pathway, which leads to Akt activation [3,31]. Akt plays a central role in promoting the survival of a wide range of cell types through various mechanisms [32,33]. Akt acts as an anti-apoptotic agent by affecting many downstream effectors of programmed cell death, such as BAD, FKHR, and caspase-3 [34,35]. Akt can also inhibit signaling pathways involved in cell damage and death, such as the c-Jun N-terminal kinase (JNK) pathway. Kim et al. described an association between Akt1 and JIP-1 (JNK binding protein 1) [36]. Akt1 inhibited JIP-1 mediated potentiation of JNK, and therefore it has been proposed that Akt1 binding to JIP-1 acts as a regulatory gate preventing the activation of JNK, which is phosphorylated under conditions of cellular stress such as excitotoxic injury [36].

Over the past years, it has been demonstrated that the PI3K/Akt pathway plays a role in HC survival. Jiang et al. showed that kanamycin alters phosphoinositide signaling in the OC *in vivo* [37]. OC explants exposed to gentamicin and a PI3K inhibitor displayed increased HC damage compared to explants exposed to gentamicin alone [20]. Haake et al. demonstrated that dexamethasone protects HCs againts TNFα-initiated apoptosis via activation of PI3K/Akt and NFκB signaling [38]. Previous reports from our laboratory are in line with these observations [8]. We found that simvastatin both protected HCs from gentamicin toxicity and activated Akt signaling *in vitro* [39].

In order to further analyze the Akt signaling pathway and to evaluate the role of Akt isoforms, we chose to use isoform-specific Akt knockout mice. The use of these mice offers advantages over the use of specific inhibitors. First, even a highly specific inhibitor will influence other signaling pathways. Second, these mice permit the assessment of specific Akt isoforms for which no inhibitor is available. Finally we can assess the hearing level in the wild-type and knockout mice. Using these mice we were able to define isoform functional specificity within the cochlea.

We found the same HC sensitivity to gentamicin in wild-type mice, mice harboring one functional copy of Akt1 or Akt1 knockout mice, suggesting Akt1 is not involved in HC survival. This is in contrast to other cell types in the body, for which higher rates of apoptosis have been observed in Akt1 knockout mice [40]. However, it should be noted that only one Akt1^-/-^ mouse could be analyzed because breeding of Akt1^-/-^ mice is quite difficult [24,27,41]. The majority of Akt1 ^-/-^ mice die within 4 days after birth [41]. In contrast, we observed enhanced HC loss in OC explants from Akt2 knockout mice exposed to gentamicin compared to wild-type mice. An intermediate level of HC damage was observed in explants from mice with one functional copy of Akt2, indicating both haploinsufficiency and HC dependence upon the level of Akt2. Interestingly, no significant difference in gentamicin-induced HC loss between Akt3^-/-^ and Akt3^+/-^ mice was observed (p = 0.0638). This indicates that Akt3 is reduced to a level that shows no additional HC protection in gentamicin-induced HC damage in mice with one functional copy of Akt3. In addition, we observed significantly greater HC loss after gentamicin exposure in OC explants from Akt2/Akt3 double knockout animals compared to Akt2 or Akt3 single knockout animals. These data strongly argue that Akt2 and Akt3 are involved in HC survival signaling. Moreover, they suggest that the level of both these isoforms is critical to HC responses to stress. From this finding, we can also propose that one isoform (Akt2 or Akt3) can partially compensate for the loss of the other isoform. The finding that Akt2 and Akt3 are involved in HC survival signaling, while Akt1 is not, is at first glance surprising. In the literature, Akt2 has mainly been linked to glucose homeostasis, while Akt3 seems to be involved in brain development. However, our findings are in line with observations in the eye. It has been shown that Akt2 but not Akt1 has a neuroprotective role in photoreceptor survival and maintenance [42]. Akt3 was not assessed in this study of the retina.

We determined hearing levels in our wild-type and knockout strains using ABR audiometry. We found significant hearing loss in Akt1 knockout and Akt2/Akt3 double knockout animals compared to wild-type, or to Akt2 and Akt3 single knockout mice. Interestingly, our data did not show any differences in the latencies of the evoked waves (data not shown), suggesting that the auditory stimuli can seemingly travel normally along the successive nuclei of the central auditory pathway, once the decibel levels surpass the elevated threshold. These findings indicate that hearing loss in Akt1 knockout and Akt2/Akt3 double knockout animals is due to cochlear damage. However, histological analysis of these animals revealed no pathology in the microanatomy of the cochlea at the light microscopic level. What might be the role of the Akt isoforms in normal hearing? One possibility is that Akt isoforms influence synaptic transmission between HCs and SG neurons. In other systems, it has been shown that the PI3K-Akt signaling pathway can modulate synaptic plasticity [43,44]. To further elucidate the nature of hearing loss in Akt knockout mice, future studies are needed.

In summary, we have shown that all three Akt isoforms are expressed in the cochlea. Moreover, we provide evidence that Akt2 and Akt3 are involved in HC survival in response to aminoglycoside ototoxic stress, while Akt1 is not. Finally, we show that Akt1 knock out and Akt2/Akt3 double knockout mice display significant hearing loss, indicating a role for all three isoforms in normal hearing.
